# BALB/c and C57BL/6 Mice Cytokine Responses to *Trypanosoma cruzi* Infection Are Independent of Parasite Strain Infectivity

**DOI:** 10.3389/fmicb.2018.00553

**Published:** 2018-03-26

**Authors:** Bianca L. Ferreira, Éden R. Ferreira, Marlon V. de Brito, Bruno R. Salu, Maria L. V. Oliva, Renato A. Mortara, Cristina M. Orikaza

**Affiliations:** ^1^Departamento de Microbiologia, Imunologia e Parasitologia, Escola Paulista de Medicina, Universidade Federal de São Paulo, São Paulo, Brazil; ^2^Departamento de Bioquímica, Escola Paulista de Medicina, Universidade Federal de São Paulo, São Paulo, Brazil

**Keywords:** Chagas’ disease, *Trypanosoma cruzi*, immune response, cytokines, mice

## Abstract

*Trypanosoma cruzi* is the etiologic agent of Chagas’ disease, which affects 6–7 million people worldwide. Different strains of *T. cruzi* present specific genotypic and phenotypic characteristics that affect the host–pathogen interactions, and thus, the parasite has been classified into six groups (TcI to TcVI). *T. cruzi* infection presents two clinical phases, acute and chronic, both with distinct characteristics and important participation by the immune system. However, the specific contributions of parasite and host factors in the disease phases are not yet fully understood. The murine model for Chagas’ disease is well-established and reproduces important features of the human infection, providing an experimental basis for the study of host lineages and parasite strains. Thus, we evaluated acute and chronic infection by the G (TcI) and CL (TcVI) strains of *T. cruzi*, which have distinct tropisms and infectivity, in two inbred mice lineages (C57BL/6 and BALB/c) that display variable degrees of susceptibility to different *T. cruzi* strains. Analysis of the parasite loads in host tissues by qPCR showed that CL strain established an infection faster than the G strain; at the same time, the response in BALB/c mice, although diverse in terms of cytokine secretion, was initiated earlier than that in C57BL/6 mice. At the parasitemia peak in the acute phase, we observed, either by confocal microscopy or by qPCR, that the infection was disseminated in all groups analyzed, with some differences concerning parasite tropism; at this point, all animals responded to infection by increasing the serum concentrations of cytokines. However, BALB/c mice seemed to better regulate the immune response than C57BL/6 mice. Indeed, in the chronic phase, C57BL/6 mice still presented exacerbated cytokine and chemokine responses. In summary, our results indicate that in these experimental models, the deregulation of immune response that is typical of chronic Chagas’ disease may be due to control loss over pro- and anti-inflammatory cytokines early in the acute phase of the disease, depending primarily on the host background rather than the parasite strain.

## Introduction

*Trypanosoma cruzi* (*T. cruzi*), a flagellate protozoan, is the etiological agent of Chagas’ disease. Approximately 6–7 million people are infected worldwide, mostly in Latin America, although the incidence has increased on other continents (WHO, 2017). In Brazil, Chagas’ disease caused 76% of the deaths due to neglected tropical diseases between 2000 and 2011 (Martins-Melo et al., 2016).

The progression and severity of Chagas’ disease vary according to individual and geographic region and also depend on parasite tissue tropism (Melo and Brener, 1978). These differences may rely on genotypic and phenotypic characteristics, particular for each *T. cruzi* isolate, interfering in host–parasite interaction. *T. cruzi* strains are distributed into six distinct groups (TcI to TcVI) based on the identification of six discrete typing units (DTUs) (Zingales et al., 2009).

Chagas’ disease has two clinical phases, acute and chronic. The acute phase lasts approximately 2 months and is usually asymptomatic or presents non-specific symptoms. In the chronic phase, patients may not experience symptoms for decades, but nearly 30% suffer from cardiomyopathy, dysfunction in the digestive tract (megaesophagus/megacolon) or alterations in the nervous system (Taniwaki et al., 2005; Rassi et al., 2010; WHO, 2017). However, controversies regarding the causes of disease manifestations in the chronic phase can be found in the literature (Nagajyothi et al., 2012). There are two main hypotheses. The first hypothesis supposes that tissue damage is primarily caused by an autoimmune response due to the molecular mimicry of host antigens by the parasite, while the second hypothesis proposed that parasite persistence in host tissues causes inflammatory responses that lead to tissue damage (Gutierrez et al., 2009; Cunha-Neto et al., 2011).

Involvement of immune system is essential in both phases of Chagas’ disease. In the acute phase, a vigorous immune response suppresses parasitemia and parasite spread; in the chronic phase, however, different response profiles are related to the asymptomatic form or the manifestation of chronic symptoms (reviewed in Boscardin et al., 2010; Basso, 2013). Several aspects of the immune response are regulated by cytokines, a heterogeneous group of soluble proteins synthesized by distinct cell types, and by chemokines, a group of cytokines responsible for cell recruitment. Although the activity of cytokines usually remains restricted to their production site, when large amounts are synthetized, they can be released into the bloodstream and present endocrine activity (Clark-Lewis et al., 1995; Dinarello, 2007). In chronic Chagas’ disease, plasma cytokines are apparently related to disease severity, either with clinical symptoms or in indeterminate phase (Sousa et al., 2014). Different cytokine expression profiles have also been related to the susceptibility to Chagas disease in the acute phase (Roggero et al., 2002). Similarly, chemokines participate in the immune response and the development of Chagas’ pathology (Aliberti et al., 1999; Cunha-Neto et al., 2009).

The murine experimental model reproduces human Chagas’ disease in many ways. For instance, mice immune response and the cytokine expression levels in the acute and chronic phases are similar to human disease. BALB/c and C57BL/6 inbred mice present different degrees of susceptibility and different resistance patterns depending on the *T. cruzi* strain. It is known that CL strain (TcVI) is highly infective in both C57BL/6 and BALB/c mice and in cell cultures (Gonçalves da Costa et al., 2002); on the other hand, the G strain (TcI) is less infective in cell cultures and might generate undetectable parasitemia levels in mice (Yoshida, 1983; Rodrigues et al., 2012). Thus BALB/c and C57BL/6 inbred mice infected with *T. cruzi* CL and G strains were used to evaluate aspects of Chagas’ disease in the acute and chronic phases, considering that the heterogeneity of the disease clinical course results from the interaction of parasite and host factors (Girones and Fresno, 2003; Dutra et al., 2009). In each group, the parasite load in host tissues was analyzed at different time-points by qPCR, the spread of the parasite was imaged by confocal microscopy, and serum levels of cytokines were measured. The results obtained in this study suggest that although there are differences in *T. cruzi* tropism according to parasite strains, the immune responses along the infection course depend primarily on the host background.

## Materials and Methods

### Ethics Statement

All animal procedures used in this study were approved by the Committee on Ethics of Animal Experiments of the Universidade Federal de São Paulo (CEUA/UNIFESP – number 33710910/14). The experiments were conducted under the Brazilian National Committee on Ethics Research (CONEP) ethical guidelines, which are in accordance with international standards (CIOMS/OMS, 1985).

### Parasites

We used G (TcI) (Yoshida, 1983) and CL (TcVI) (Brener and Chiari, 1963) *T. cruzi* strains, wild-type or transfected with GFP (G strain) (Cruz et al., 2012) or DsRed (CL strain) (Ferreira et al., 2016) in pTREX plasmids. Tissue-culture derived trypomastigotes (TCTs), analogous to bloodstream trypomastigotes, were obtained from the supernatant of infected Vero cells (Instituto Adolfo Lutz, São Paulo, SP, Brazil) that were grown in RPMI 1640 medium (Vitrocell, Embriolife, Brazil) supplemented with 2% fetal bovine serum (FBS) (Gibco, Thermo Fisher Scientific, United States), 10 μg/mL streptomycin, 100 U/mL penicillin and 40 μg/mL gentamicin (antibiotics from Sigma-Aldrich, United States) at 37°C and 5% CO_2_.

### Mice and *T. cruzi* Infection

BALB/c and C57BL/6 female mice ranging from 6 to 8 weeks old were infected intraperitoneally with 10^6^ TCTs in 0.5 ml PBS; the control animals were injected with a PBS solution prepared after centrifugation of the supernatant of Vero cells. Parasitemia was monitored as described by Brener (1962) in both BALB/c and C57BL/6 mice using the CL strain as a reference, since the G strain did not present detectable parasitemia levels. Three time points were then determined for animal euthanasia: 2 days post infection (dpi), at the beginning of infection; 8 dpi, at the peak of parasitemia in the acute phase; and 90 dpi, during the chronic infection.

### DNA Extraction and Quantitative Real-Time PCR

Primers for a repetitive sequence of *T. cruzi* DNA (TCZ-F 5′-GCTCTTGCCCACAMGGGTGC-3′, M = A or C and TCZ-R 5′-CCAAGCAGCGGATAGTTCAGG-3′), which amplify a 182-bp sequence, and primers for murine TNF (TNF-5241 5′-TCCCTCTCATCAGTTCTATGGCCCA-3′ e TNF-5411 5′-CAGCAAGCATCTATGCACTTAGACCCC-3′), which amplify a 170-bp sequence, were synthesized, as previously described for *T. cruzi* tissue quantification (Cummings and Tarleton, 2003). BALB/c and C57BL/6 mice infected with *T. cruzi* G or CL strains were euthanized and the hearts, livers, intestines and spleens were removed, sectioned and immediately subjected to DNA extraction using the DNeasy^®^ Blood & Tissue Kit (Qiagen, Germany), according to manufacturer’s instructions. The reactions were performed with 20 ng of genomic DNA, 10 μL of SYBR^®^ Select Master Mix (Applied Biosystems, United States) and 0.35 μM each forward and reverse primer in the StepOnePlus System (Applied Biosystems, United States). Each plate contained a standard curve and two negative controls, a control with no template and an uninfected tissue control. The samples were quantified in duplicate, and data were normalized by the TNF gene quantity in each sample (Cummings and Tarleton, 2003).

The standards for the PCR reactions were generated by adding 10^6^ *T. cruzi* epimastigotes to 20 mg of tissue extracted from uninfected mice. After DNA extraction, the material was 10-fold serially diluted with 20 μg/ml uninfected tissue DNA. A standard curve was then generated from 0.1 to 10000 parasite equivalents per 20 ng of total DNA, as previously described (Cummings and Tarleton, 2003).

### Tissue Imaging

The spleens, livers, intestines, and hearts were removed from mice infected with G-GFP or CL-DsRed fluorescent parasites. The organs were placed onto a cold paraffin surface, and slices were cut with two parallel GEM single-edge blades, immersed in PBS containing 1 μM Hoechst 33342 (Invitrogen, Carlsbad, CA, United States) to label nuclei and then transferred to glass-bottom dishes (MatTek Corporation, Ashland, MA, United States) in RPMI-1640 medium (Vitrocell, Embriolife, Brazil) supplemented with 10% FBS (Gibco, Thermo Fisher Scientific, United States). *Ex vivo* specimens were transferred to a stage-top incubator at 37°C and 5% CO_2_ with controlled humidity (Tokai Hit, Japan) on a Leica SP5 TS confocal microscope and imaged with a 63 × NA 1.40 oil-immersion objective using the resonant scanner (8000 Hz) mode, as described (Ferreira et al., 2016). Final imaging processing was performed using Imaris software 7.0 (Bitplane).

### Quantitation of Serum Cytokines and Chemokines

Whole blood was obtained by cardiac puncture in infected and control mice and was collected into tubes containing protease inhibitors, including 10 mM EDTA (Thermo Fisher Scientific, United States), 9 μM aprotinin (Sigma-Aldrich, United States) and 10 μM E-64 (Sigma-Aldrich, United States). The tubes were then centrifuged at 3400 *g* for 5 min at 4°C, and each serum sample was separated into two 110-μL aliquots, quick frozen in liquid nitrogen and stored at -80°C. These samples were used to quantify the levels of chemokines and cytokines using the following kits according to the manufacturer’s instructions: MILLIPLEX^®^MAP Mouse Cytokine/Chemokine Magnetic Bead Panel (Merck Millipore, Germany) for TNF-α, INF-γ, IL-2, IL-1α, IL-1β, IL-9, IL-17, IL-6, IL-5, IL-4, IL-10, KC (CXCL1), MIG (CXCL9), IP10 (CXCL10), MCP-1 (CCL2), MIP-1α (CCL3), MIP-1β (CCL4), RANTES (CCL5), and Eotaxin (CCL11) quantitation and MILLIPLEX^®^MAP TGF-β1, 2, 3 Magnetic Bead Kit (Merck Millipore, Germany) for TGF-β1 quantitation in the MAGPIX^®^ system (Merck Millipore, Germany). All samples were analyzed in duplicate.

### Statistical Analysis

The experiments were performed with three mice per group, and the results are presented as the mean ± standard deviation or a box plot. The differences were considered statistically significant when *p* < 0.05 by one-way (cytokines quantitation) or two-way (qPCR) ANOVA followed by Bonferroni multiple comparisons test in Prism 6.0 software (GraphPad, United States).

## Results

### Parasite Loads in Mouse Organs Depend on *T. cruzi* Strain

**FIGURE 1:**
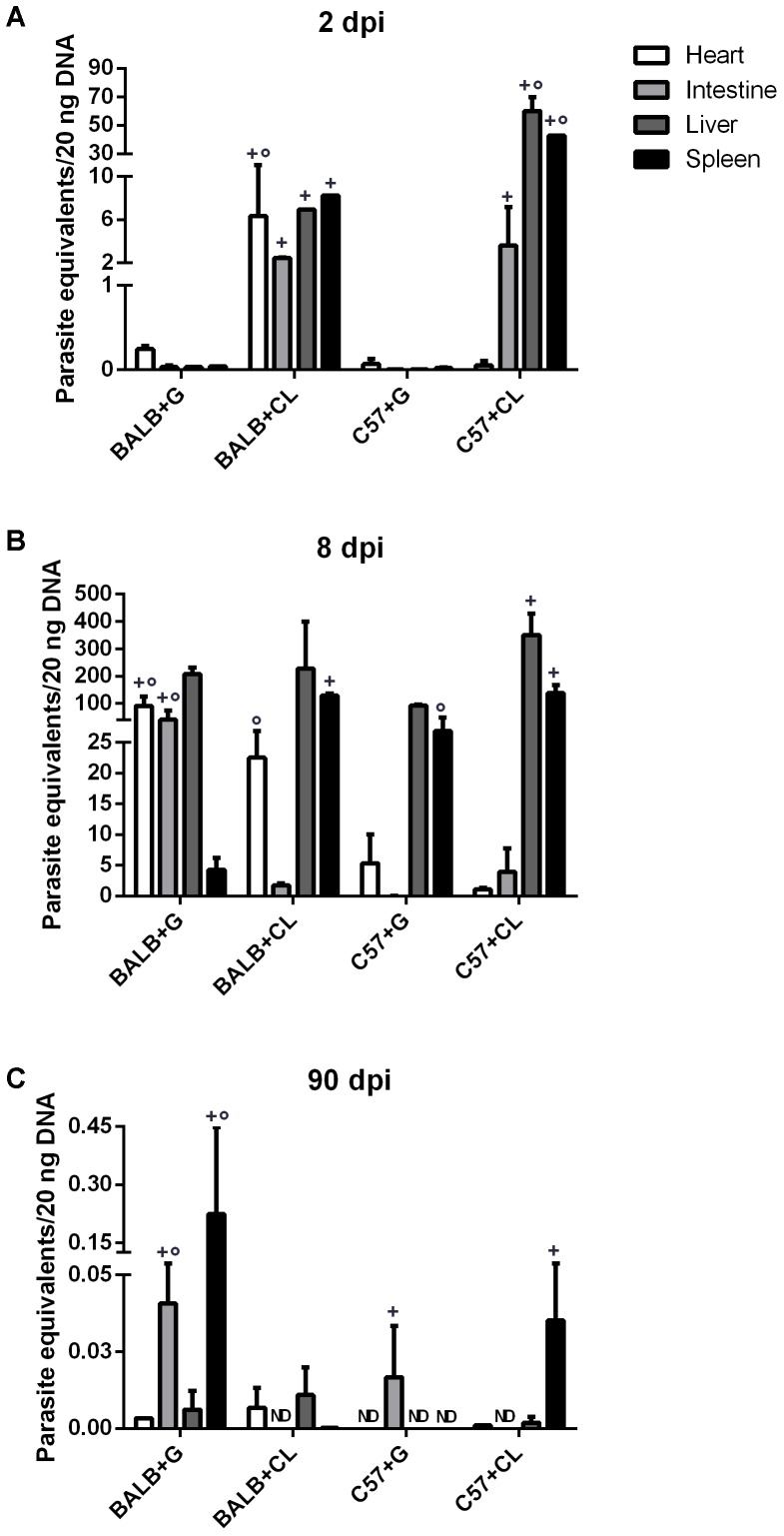
Parasite-equivalents in murine tissues quantified by qPCR. BALB/c and C57BL/6 mice infected with G or CL strains were euthanized at 2 **(A)**, 8 **(B)**, or 90 dpi **(C)**. Genomic DNA was then extracted from the hearts, intestines, livers, and spleens for quantification of parasite-equivalents by qPCR. Comparisons were made between organs separately. + indicates a statistically significant difference between parasite strains in infected mice from a single lineage, ° indicates a statistically significant difference between mice lineages when infected with the same parasite strain. Both + and ° indicate *p* < 0.05. ND, not detected.

Parasite loads were quantified in the spleens, hearts, livers, and intestines from BALB/c and C57BL/6 mice at each infection time-point. The parasites were initially found in both mice infected with CL strain at 2 dpi. At this time point parasites were detected in all analyzed organs from CL-strain infected BALB/c mice. In C57BL/6 mice infected with CL, the livers and spleens held the largest quantity of parasites, while few parasites were found in the hearts. However, in animals infected with G strain, parasites were detected in much lower amounts than were observed in those infected with CL strain (**Figure 1A**).

**FIGURE 2:**
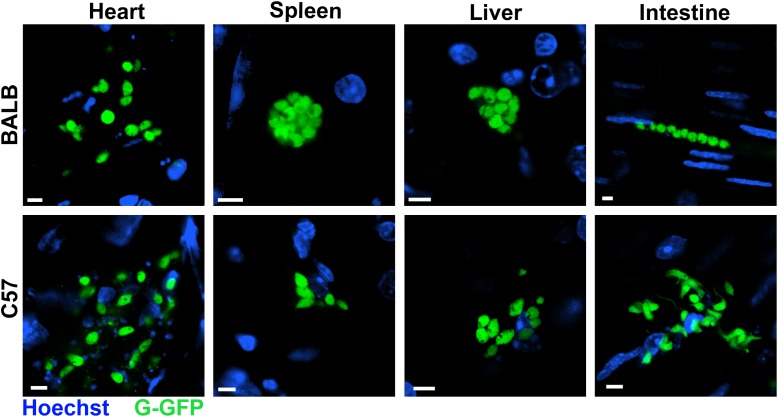
*Trypanosoma cruzi* distributed in murine organs in the acute phase. Hearts, spleens, livers, and intestines of BALB/c and C57BL/6 mice infected with G-GFP (green) were sectioned, incubated with Hoechst to label the nuclei (blue) and maintained *ex vivo* in dishes with culture medium for imaging by confocal microscopy. Parasite nests were found in all analyzed organs at 8 dpi. Bars = 5 μm.

At 8 dpi, parasites were detected mainly in the livers and spleens of all infected mice, except in BALB/c infected with G strain, in which the quantity of parasites in the spleen was lower. Additionally, in this group there were many parasites in the heart and intestine. On the other hand, BALB/c mice infected with the CL strain showed a high parasite load in the spleen, liver, and heart, but not in intestine. In C57BL/6 mice infected with G strain, the numbers of parasites in the spleen and liver were lower than in those infected with CL strain (**Figure 1B**). Overall, in the acute phase, parasites were distributed in different organs of all mice. Similar results were observed by confocal microscopy in live tissues extracted from mice infected with G strain-GFP, where parasite nests were found in all analyzed tissues (**Figure 2**). The same procedure were performed in CL-DsRed infected mice, but due to weak fluorescence we could not obtain representative images. Nonetheless, images of CL-DsRed infected tissues (mice) can be found in our previous work (Ferreira et al., 2016).

At 90 dpi, (chronic phase) *T. cruzi* detection was clearly lower than at previous infection time points, and all mice succeeded in controlling parasitemia, since parasite loads were not higher than 1 parasite equivalent per 20 ng of total DNA in all tissues. In BALB/c mice infected with G strain, parasites were especially found in the intestine and spleen; and in those infected with CL strain, parasites were found in similar amounts in the heart and liver. In C57BL/6 mice infected with the G strain, *T. cruzi* DNA was found exclusively in the intestine; and in mice infected with the CL strain, there was parasite DNA in the spleen (**Figure 1C**).

### Quantification of Cytokines and Chemokines Levels in Serum

**FIGURE 3:**
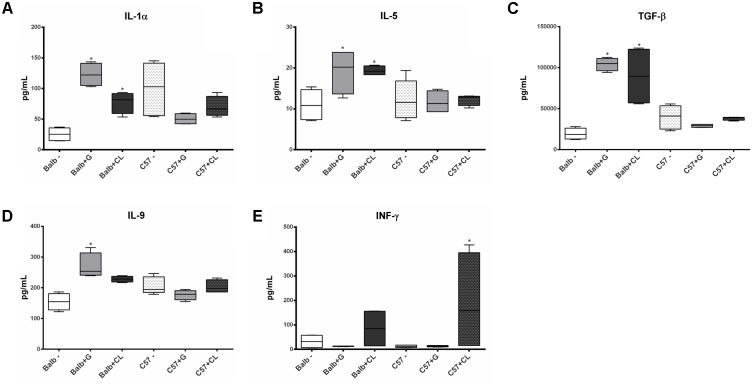
Cytokines with altered serum levels in infected mice at 2 dpi. Serum samples were obtained from C57BL/6 and BALB/c mice uninfected or infected with G or CL strains at 2 dpi for the quantification of cytokines levels. Box plots correspond to the concentrations of IL-1α **(A)**, IL-5 **(B)**, TGF-β **(C)**, IL-9 **(D)**, and INF-γ **(E)**, which were different in some groups from the control mice at this time point. ^∗^ indicates a significant difference (*p* < 0.05) between infected and uninfected mice [control (–)].

At 2 dpi, BALB/c mice infected with both G and CL strains presented increased concentrations of IL-1α (**Figure 3A**), IL-5 (**Figure 3B**), and TGF-β (**Figure 3C**), IL-9 level was also increased, but only in those infected with G strain (**Figure 3D**). At this time point, no variation in cytokines in C57BL/6 mice compared to control group were found, except for INF-γ, which was increased in CL-infected animals (**Figure 3E**). Although not statistically significant, we observed an increase in the level of this cytokine in BALB/c mice infected with CL strain.

**FIGURE 4:**
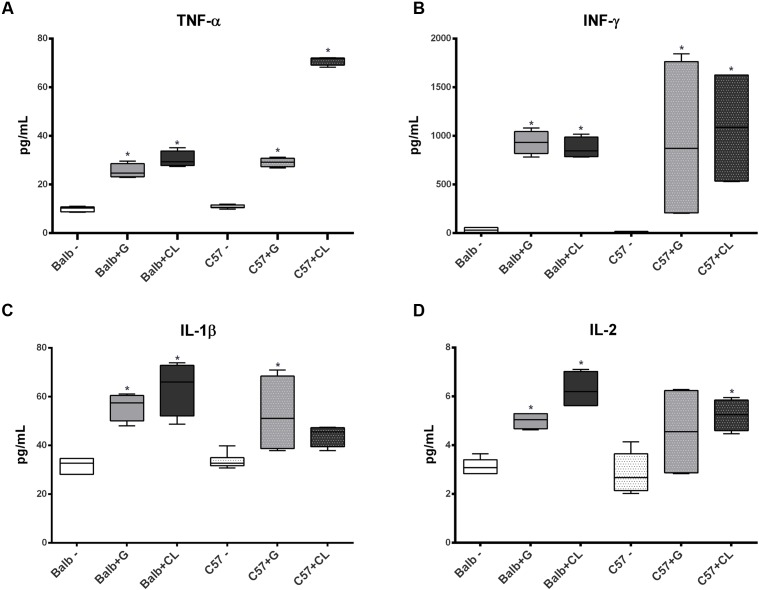
Th1 cytokine levels altered in infected mice sera at 8 dpi. The concentrations of TNF-α **(A)**, INF-γ **(B)**, IL-1β **(C)**, and IL-2 **(D)** cytokines in serum samples from C57BL/6 and BALB/c mice infected with G or CL strains differed from those in the serum samples of control mice at 8 dpi. The data are presented as box plots, and ^∗^ indicates a significant difference (*p* < 0.05) between the infected and uninfected mice [control (–)].

**FIGURE 5:**
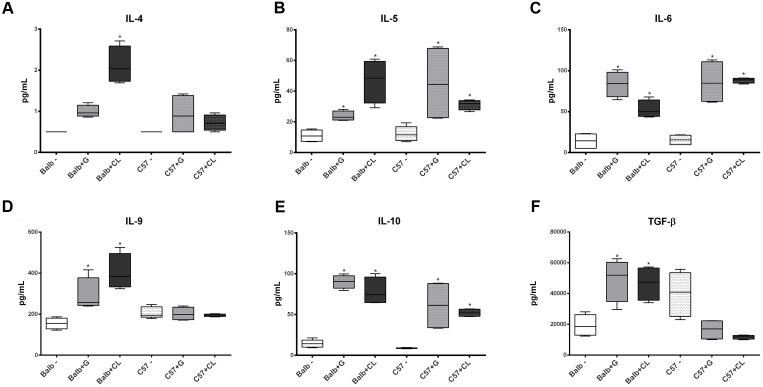
Alterations in Th2 and anti-inflammatory cytokine levels at 8 dpi. At 8 dpi, the levels of the Th2 cytokines IL- 4 **(A)**, IL-5 **(B)**, and IL-6 **(C)**, as well as those of IL-9 **(D)** and anti-inflammatory cytokines IL-10 **(E)** and TGF-β **(F)** were quantified in the serum from C57BL/6 and BALB/c uninfected mice or those infected with G or CL strains and were found to be different. The data are presented as box plots, and ^∗^ indicates a significant difference (*p* < 0.05) between the infected and uninfected mice [control (–)].

**FIGURE 6:**
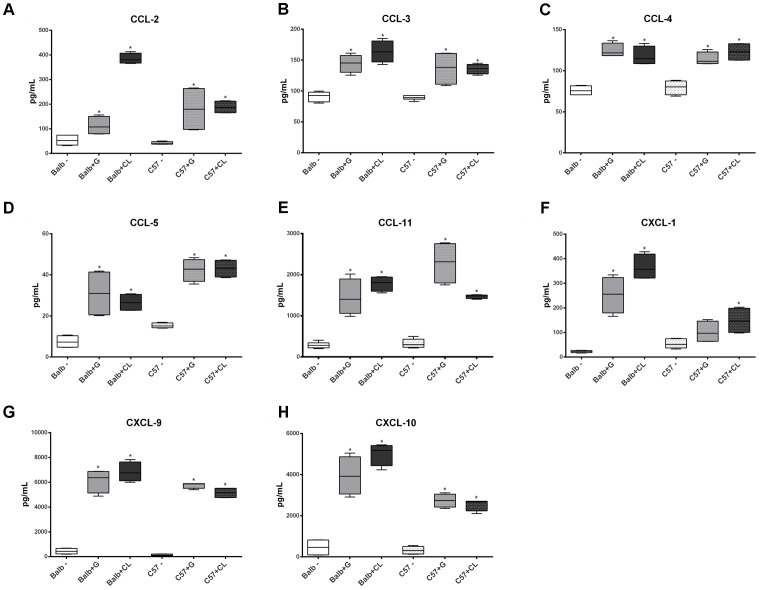
Altered concentrations of serum chemokines in infected mice at 8 dpi. Serum samples were obtained from healthy C57BL/6 and BALB/c mice and from those infected with G or CL strains at 8 dpi for the quantification of chemokines levels. The concentrations of CCL-2 **(A)**, CCL-3 **(B)**, CCL-4 **(C)**, CCL-5 **(D)**, CCL-11 **(E)**, CXCL-1 **(F)**, CXCL-9 **(G)**, and CXCL-10 **(H)** were measured and differed from those of the control. The data are summarized as box plots, and ^∗^ indicates a significant difference (*p* < 0.05) between the infected and uninfected mice [control (–)].

At 8 dpi, concentrations of several cytokines and chemokines were increased in both mice lineages infected with *T. cruzi* G and CL strains (**Figures 4–6**). TNF-α, INF-γ, IL-1β, and IL-2, Th1 cytokines, which are related to host resistance and protection, were increased in almost all experimental groups (**Figure 4**). IL-1β and IL-2 concentrations in C57BL/6 mice infected with CL and G strains, respectively, did not show significant difference when compared to those of healthy mice but were present in slightly higher amounts (IL-1β: uninfected mice 33.54 ± 3.21 pg/mL, C57BL/6 + CL strain 44.07 ± 4.35 pg/mL; IL-2: uninfected mice 2.86 ± 0.86 pg/mL, C57BL/6 + G strain 4.55 ± 1.89 pg/mL). IL-4, IL-5, and IL-6, which are Th2-related cytokines, were also quantified; IL-4 concentration changed only in CL-infected BALB/c mice (**Figure 5A**), while serum IL-5 and IL-6 levels increased in BALB/c and C57BL/6 mice infected with both strains (**Figures 5B,C**). The proinflammatory cytokine IL-9 was increased only in BALB/c mice independent of parasite strain (**Figure 5D**). Regarding to anti-inflammatory cytokines, IL-10 concentration was increased in all mice infected with any parasite strain (**Figure 5E**), while level of TGF-β was increased only in BALB/c infected mice (**Figure 5F**). Additionally, serum concentrations of CCL-2, CCL-3, CCL-4, CCL-5, CCL-11, CXCL-1, CXCL-9, and CXCL-10 chemokines were increased in both BALB/c and C57BL/6 infected with G and CL strains (**Figure 6**), although level of CXCL-1 in G-infected C57BL/6 mice was not significantly different from that of the non-infected group. In general, at this time point, all animals responded to infection with the release of cytokines, regardless of mouse lineage or *T. cruzi* strain, which is expected since in all groups the parasites were distributed in different tissues.

**FIGURE 7:**
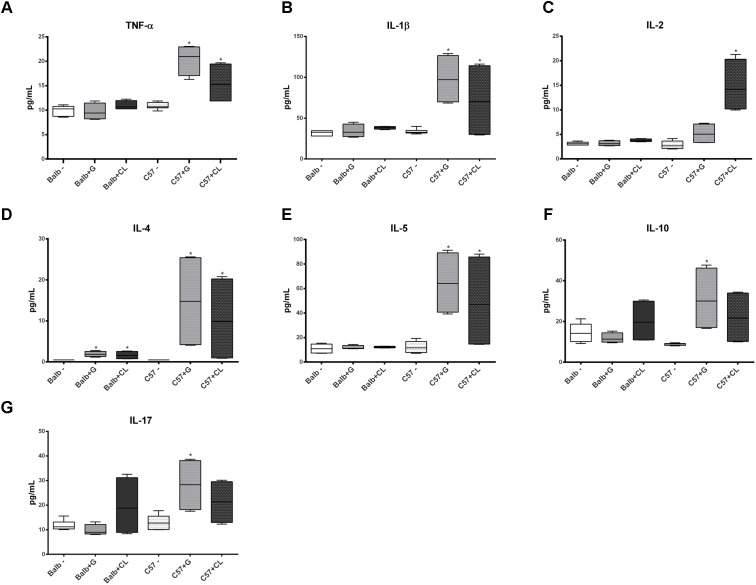
Altered levels of cytokines in infected mice at 90 dpi. The concentrations of TNF-α **(A)**, IL-1β **(B)**, IL-2 **(C)**, IL-4 **(D)**, IL-5 **(E)**, IL-10 **(F)**, and IL-17 **(G)** were quantified in the serum from C57BL/6 and BALB/c uninfected mice and from those infected with G or CL strains at 90 dpi, and their concentrations were found to be different from those of the control. The data are represented as box plots, and ^∗^ indicates a significant difference (*p* < 0.05) between the infected and uninfected mice [control (–)].

**FIGURE 8:**
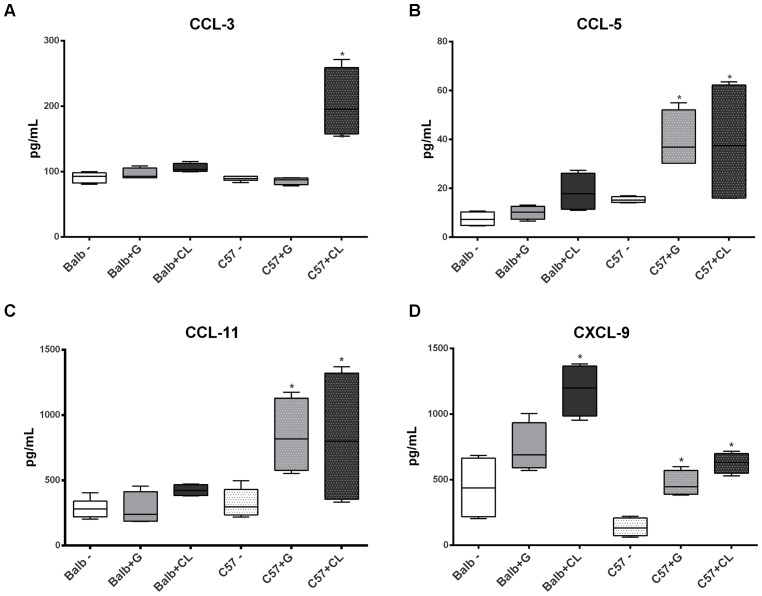
Altered levels of serum chemokines at 90 dpi. Box plots represent the concentrations of CCL-3 **(A)**, CCL-5 **(B)**, CCL-11 **(C)**, and CXCL-9 **(D)**, which were quantified in the serum from C57BL/6 and BALB/c uninfected mice or those infected with G or CL strains, and the concentrations differed from those of the control at 90 dpi. ^∗^ indicates a significant difference (*p* < 0.05) between the infected and uninfected mice [control (–)].

Interesting differences were observed between groups in chronic phase at 90 dpi. The levels of several cytokines were increased in C57BL/6 mice serum independent of *T. cruzi* strain, such as TNF-α, IL1-β, IL-4, IL-5, CCL-5, CCL-11, and CXCL-9 (**Figures 7**, **8**); the levels of IL-10 and IL-17 were only increased with G-strain infection (**Figures 7F,G**), and the levels of IL-2 and CCL-3 were only increased in mice infected with the CL strain (**Figures 7C**, **8A**). On the other hand, only the IL-4 cytokine concentration was altered in BALB/c mice infected with each *T. cruzi* strain (**Figure 7D**), and in those infected with the CL strain there was an increase in the quantity of CXCL-9 (**Figure 8D**). All cytokine levels were measured in all time points, but those that did not differ from the levels in the control groups are not shown.

## Discussion

Chagas’ disease has acute and chronic phases. In the chronic phase, patients may be asymptomatic or exhibit cardiomyopathy, dysfunction in the digestive tract or alterations in the nervous system (Taniwaki et al., 2005; Rassi et al., 2010). The host immune system plays key roles during entire infection course. In acute phase, it acts to control the infection and parasite spread, while in chronic phase, different responses are related to manifestation or absence of symptoms (Boscardin et al., 2010; Basso, 2013). There are two main hypotheses concerning the primary causes of the chronic manifestation: one hypothesis is related to a host autoimmune response and the other supposes that the inflammatory response is sustained by the persistence of parasites in tissues (Gutierrez et al., 2009; Cunha-Neto et al., 2011). Thus, considering the importance of both parasite and host factors for the disease progress, we used two host lineages infected with distinct *T. cruzi* strains to evaluate aspects of acute and chronic phases.

**FIGURE 9:**
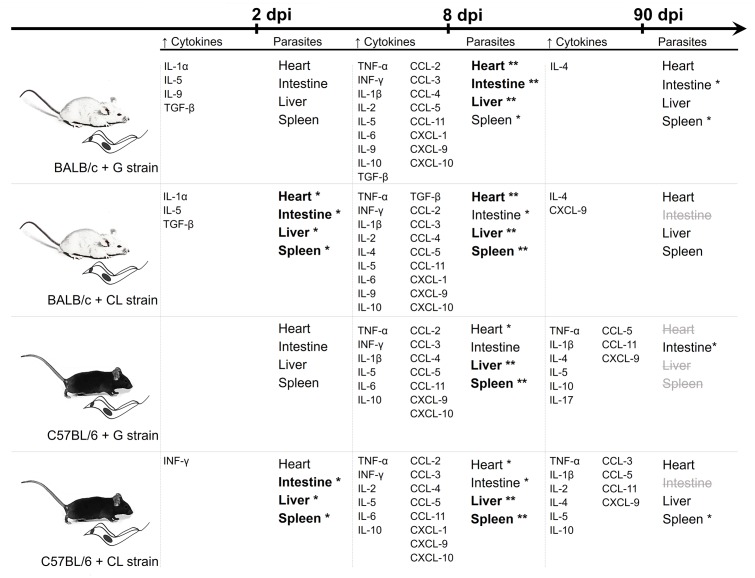
Summary of the data from the quantification of serum cytokines and parasite loads. The results of the quantification of serum cytokines levels and parasite loads are summarized in parallel at each time point. The cytokines and chemokines were included when the concentrations were significantly different from those of the uninfected mice. Bold fonts and asterisks highlight organs with the highest parasite quantities at each time point, while strikethrough gray fonts are used when *T. cruzi* DNA was not detectable. The data observed together suggests that while there are differences in *T. cruzi* tropism due to parasite strains, the release of cytokines seems to depend primarily on the host background.

When results from quantification of parasite loads and concentrations of serum cytokines are collectively reviewed (**Figure 9**), it is reasonable to conclude that, although parasite tropism was influenced by strain, the release of host cytokines seemed to be related mainly to the mouse lineage. While at 8 dpi, parasites were spread through the host tissues, and all animals showed increased levels of cytokines, earlier, at 2 dpi, infected BALB/c mice showed early release of some cytokines, which did not occur in C57BL/6 mice. In chronic phase (90 dpi), although all animals were efficient in controlling parasite spread, C57BL/6 mice had higher amounts of serum cytokines, independent of *T. cruzi* strain.

Detailed analysis of the acute phase initial time point (2 dpi) demonstrated that differences in infectivity of parasite strains were relevant for an early infection establishment, since CL strain was detected within the organs of infected mice at 2 dpi. In fact, strain related characteristics may influence the disease development (reviewed by Dutra et al., 2014). Interestingly, at this time point, IFN-γ was detectable in animals infected with CL parasites. As an important cytokine for the activation of innate response and parasite destruction (Torrico et al., 1991; Holscher et al., 1998), increased IFN-γ expression may be a simple response to high parasite load. However, other cytokines were increased only in BALB/c mice, unrelated to the parasite strain. A study with the same mice lineage investigated why *in vitro* high infective G strain amastigotes fail to establish *in vivo* parasitemia, and the authors concluded that an early response of inflammatory cytokines such as IL-12 and INF-γ were important in controlling the infection (Rodrigues et al., 2012). Although the work of Rodrigues did not evaluate the alterations in IL-1β, IL-5, TGF-β, and IL-9 cytokine levels, it is possible to infer that early release of cytokines is relevant to BALB/c mice response and it is related to host lineage, since this pattern was not observed in C57BL/6 mice.

In experimental studies, resistance to *T. cruzi* infection was related to Th1 cytokine profile, such as IFN- γ, TNF-α, and IL-1 (Silva et al., 1995; Sanoja et al., 2013), and Th2, which is required for tissue protection (Basso, 2013). In our study, virtually all animals showed increased levels of TNF-α, INF-γ, IL-1β, IL-2, IL-5, and IL-6 at 8 dpi. The importance of these cytokines in the response to infection may be seen in recent studies in which the inhibition of IL-18 or 5-lipoxygenase improved the resistance of mice to parasites in acute phase, with an increase in levels of cytokines such as IL-12, INF-γ, IL-1β, and IL-6 and a decrease in the level of IL-10 (Canavaci et al., 2014; Esper et al., 2014). Similar to TGF-β, IL-10 is an anti-inflammatory cytokine related to infection susceptibility (Silva et al., 1991; Canavaci et al., 2014; Esper et al., 2014). Our results revealed that all animals had increased levels of IL-10 in their serum, and only BALB/c mice presented an increased release of TGF-β. These cytokines are important in the control of immune response, for instance, a study described that deregulation of pro-and anti-inflammatory cytokines was implicated in disease lethality during acute phase. Susceptible mice in acute phase had even greater release of TNF-α than the resistant lineage, but a lower release of IL-10 (Roggero et al., 2002). Thus, the fact that all mice showed increased IL-10 levels but only BALB/c showed increased TGF-β levels at this infection time point may imply that these mice presented a more regulated and efficient response than those of C57BL/6 lineage. Regarding the chemokines, diverse studies in humans and mice have indicated that CXCL1, CXCL9, CXCL10, CCL2, CCL3, CCL4, and CCL5 are important in controlling the infection during the acute phase (Aliberti et al., 1999; Hardison et al., 2006; Cunha-Neto et al., 2009; Paiva et al., 2009), and the levels of virtually all of them were increased in both BALB/c and C57BL/6 mice. In addition, both mouse lineages had an increase in the level of CCL11 at 8 dpi, and although there are not many studies addressing the relationship of this chemokine to *T. cruzi* infection response, it has been described that CCL11 mRNA expression is increased in cardiomyocytes infected with the parasite (Udoko et al., 2016), and this chemokine has been related to myocardial fibrosis (Zweifel et al., 2010). At this stage, no relation was observed between the host response and the spread of the parasite; in all cases, high parasite loads and the presence of nests were observed in different tissues, with only small differences in tropism, such as in the heart, where the parasite load was higher in BALB/c mice, especially those infected with the G strain. Therefore, it is noteworthy to mention that in a recent study, CL strain was almost undetected in the heart of orally infected mice (Rodrigues et al., 2016).

Regarding to the chronic phase, several studies in both humans and experimental models reported that the presence of an undetermined or symptomatic chronic phase is directly related to inflammatory response and the type of cytokines present in the subject. Chronic chagasic patients display predominantly cytokines such as TNF-α, INF-γ, and IL-6, and asymptomatic patients have higher amounts of IL-4, IL-10, IL-13, and TGF-β (Gomes et al., 2003; Poveda et al., 2014; Sousa et al., 2014). The present study demonstrated that at 90 dpi, BALB/c mice had alterations only in levels of IL-4 and CXCL-9 (in those infected with G strain). CXCL9 is important in the development of chronic disease (Nogueira et al., 2012), but no essential role in tissue inflammation has been observed for it (Hardison et al., 2006). Regarding to IL-4, knockout mice displayed reduced acute phase parasitemia but showed subsequent increases in inflammation compared to non-depleted mice (Soares et al., 2001), indicating that this cytokine is necessary for tissue protection in the chronic phase. On the other hand, we observed that in all C57BL/6 mice, levels of several cytokines and serum chemokines increased at this infection time point (TNF-α, IL-1β, IL-4, IL-5, CCL-5, CCL-11, and CXCL-9, in addition to IL-2, IL-10, IL-17 and CCL-3, depending on the strain of *T. cruzi*), indicating a lack of immune response control similar to that occurring in the chronic phase (Perez et al., 2011; Sousa et al., 2014). TNF-α is particularly increased in chronic patients and is considered an important indicator of cardiomyopathy (Talvani et al., 2004; Requena-Mendez et al., 2013; Keating et al., 2015). Interestingly, despite different responses observed between BALB/c and C57BL/6 mice, qPCR results showed that both were efficient to control the infection. These results indicate that persistence of parasites in the organs is not precisely related to inflammatory response. Supporting our findings, a study showed that mice infected with CL Brener had cardiac fibrosis and myocarditis, even without the presence of parasites in heart (Lewis et al., 2014). On the other hand, it is possible that C57BL/6 mice were more efficient in eliminating the parasite, since parasite amount in the organs at 90 dpi were smaller in C57BL/6 mice than in BALB/c mice (undetectable in a few cases). Nevertheless, these mice were inefficient at controlling their immune responses, which may have led to the observed cytokine profiles. This possibility is reinforced by the fact that in the acute phase, there were smaller amounts of anti-inflammatory cytokines in C57BL/6 mice than in BALB/c mice.

In experimental infection characteristics of *T. cruzi* infection differ according to mouse lineage and parasite strain (Gonçalves da Costa et al., 2002; Sanoja et al., 2013). The results presented here using isogenic BALB/c and C57BL/6 mice infected with G (Tc I, less infective) or CL (Tc VI, more infective) strains suggest that in these models differences in the immune response during disease progression is likely related to host characteristics and not to strain infectivity. Accordingly, studies in literature support the notion on the relevance of host genetic background in the manifestation of symptoms in chronic Chagas’ disease (Frade et al., 2013; Luz et al., 2016). Additionally, the exacerbated inflammatory response observed in chronic phase of the disease may be the result of a deficiency in controlling the host response (that initially leads to parasite suppression) but not necessarily due to parasite persistence in different host tissues.

## Author Contributions

RM, BF, and CO conceived the study. BF, CO, MO, and RM designed the experiments. BF, CO, MdB, and BS performed the experiments. BF, ÉF, MO, RM, and CO interpreted the results. BF, ÉF, RM and CO wrote the manuscript.

## Conflict of Interest Statement

The authors declare that the research was conducted in the absence of any commercial or financial relationships that could be construed as a potential conflict of interest.

## References

[B1] Aliberti J. C. Machado F. S. Souto J. T. Campanelli A. P. Teixeira M. M. Gazzinelli R. T. (1999). beta-Chemokines enhance parasite uptake and promote nitric oxide-dependent microbiostatic activity in murine inflammatory macrophages infected with *Trypanosoma cruzi*. *Infect. Immun.* 67 4819–4826. 10456936 10.1128/iai.67.9.4819-4826.1999 PMC96814

[B2] Basso B. (2013). Modulation of immune response in experimental Chagas disease. *World J. Exp. Med.* 3 1–10. 10.5493/wjem.v3.i1.1 24520540 PMC3905588

[B3] Boscardin S. B. Torrecilhas A. C. Manarin R. Revelli S. Rey E. G. Tonelli R. R. (2010). Chagas’ disease: an update on immune mechanisms and therapeutic strategies. *J. Cell. Mol. Med.* 14 1373–1384. 10.1111/j.1582-4934.2010.01007.x 20070438 PMC3829005

[B4] Brener Z. (1962). Therapeutic activity and criterion of cure on mice experimentally infected with *Trypanosoma cruzi*. *Rev. Inst. Med. Trop. Sao Paulo* 4 389–396. 14015230

[B5] Brener Z. Chiari E. (1963). Morphological variations observed in different strains of *Trypanosoma cruzi*. *Rev. Inst. Med. Trop. Sao Paulo* 5 220–224. 14110094

[B6] Canavaci A. M. Sorgi C. A. Martins V. P. Morais F. R. de Sousa E. V. Trindade B. C. (2014). The acute phase of *Trypanosoma cruzi* infection is attenuated in 5-lipoxygenase-deficient mice. *Mediators Inflamm.* 2014:893634. 10.1155/2014/893634 25165415 PMC4137569

[B7] Clark-Lewis I. Kim K. S. Rajarathnam K. Gong J. H. Dewald B. Moser B. (1995). Structure-activity relationships of chemokines. *J. Leukoc. Biol.* 57 703–711. 10.1002/jlb.57.5.703 7759949

[B8] Cruz M. C. Souza-Melo N. da Silva C. V. Darocha W. D. Bahia D. Araujo P. R. (2012). *Trypanosoma cruzi*: role of delta-amastin on extracellular amastigote cell invasion and differentiation. *PLoS One* 7:e51804. 10.1371/journal.pone.0051804 23272170 PMC3525664

[B9] Cummings K. L. Tarleton R. L. (2003). Rapid quantitation of *Trypanosoma cruzi* in host tissue by real-time PCR. *Mol. Biochem. Parasitol.* 129 53–59. 10.1016/S0166-6851(03)00093-8 12798506

[B10] Cunha-Neto E. Nogueira L. G. Teixeira P. C. Ramasawmy R. Drigo S. A. Goldberg A. C. (2009). Immunological and non-immunological effects of cytokines and chemokines in the pathogenesis of chronic Chagas disease cardiomyopathy. *Mem. Inst. Oswaldo Cruz* 104 (Suppl. 1), 252–258. 10.1590/S0074-02762009000900032 19753481

[B11] Cunha-Neto E. Teixeira P. C. Nogueira L. G. Kalil J. (2011). Autoimmunity. *Adv. Parasitol.* 76 129–152. 10.1016/B978-0-12-385895-5.00006-2 21884890

[B12] Dinarello C. A. (2007). Historical insights into cytokines. *Eur. J. Immunol.* 37 (Suppl. 1), S34–S45. 10.1002/eji.200737772 17972343 PMC3140102

[B13] Dutra W. O. Menezes C. A. Magalhaes L. M. Gollob K. J. (2014). Immunoregulatory networks in human Chagas disease. *Parasite Immunol.* 36 377–387. 10.1111/pim.12107 24611805 PMC4143493

[B14] Dutra W. O. Menezes C. A. Villani F. N. da Costa G. C. da Silveira A. B. Reis D. (2009). Cellular and genetic mechanisms involved in the generation of protective and pathogenic immune responses in human Chagas disease. *Mem. Inst. Oswaldo Cruz* 104 (Suppl. 1), 208–218. 10.1590/S0074-02762009000900027 19753476 PMC3285444

[B15] Esper L. Utsch L. Soriani F. M. Brant F. Esteves Arantes R. M. Campos C. F. (2014). Regulatory effects of IL-18 on cytokine profiles and development of myocarditis during *Trypanosoma cruzi* infection. *Microbes Infect.* 16 481–490. 10.1016/j.micinf.2014.03.007 24704475

[B16] Ferreira B. L. Orikaza C. M. Cordero E. M. Mortara R. A. (2016). *Trypanosoma cruzi*: single cell live imaging inside infected tissues. *Cell. Microbiol.* 18 779–783. 10.1111/cmi.12553 26639617 PMC5064609

[B17] Frade A. F. Pissetti C. W. Ianni B. M. Saba B. Lin-Wang H. T. Nogueira L. G. (2013). Genetic susceptibility to Chagas disease cardiomyopathy: involvement of several genes of the innate immunity and chemokine-dependent migration pathways. *BMC Infect. Dis.* 13:587. 10.1186/1471-2334-13-587 24330528 PMC3866603

[B18] Girones N. Fresno M. (2003). Etiology of Chagas disease myocarditis: autoimmunity, parasite persistence, or both? *Trends Parasitol.* 19 19–22. 10.1016/S1471-4922(02)00006-5 12488221

[B19] Gomes J. A. Bahia-Oliveira L. M. Rocha M. O. Martins-Filho O. A. Gazzinelli G. Correa-Oliveira R. (2003). Evidence that development of severe cardiomyopathy in human Chagas’ disease is due to a Th1-specific immune response. *Infect. Immun.* 71 1185–1193. 10.1128/IAI.71.3.1185-1193.2003 12595431 PMC148818

[B20] Gonçalves da Costa S. C. Calabrese K. S. Zaverucha do Valle T. Lagrange P. H. (2002). *Trypanosoma cruzi*: infection patterns in intact and athymic mice of susceptible and resistant genotypes. *Histol. Histopathol.* 17 837–844. 10.14670/HH-17.837 12168794

[B21] Gutierrez F. R. Guedes P. M. Gazzinelli R. T. Silva J. S. (2009). The role of parasite persistence in pathogenesis of Chagas heart disease. *Parasite Immunol.* 31 673–685. 10.1111/j.1365-3024.2009.01108.x 19825107

[B22] Hardison J. L. Wrightsman R. A. Carpenter P. M. Lane T. E. Manning J. E. (2006). The chemokines CXCL9 and CXCL10 promote a protective immune response but do not contribute to cardiac inflammation following infection with *Trypanosoma cruzi*. *Infect. Immun.* 74 125–134. 10.1128/IAI.74.1.125-134.2006 16368965 PMC1346648

[B23] Holscher C. Kohler G. Muller U. Mossmann H. Schaub G. A. Brombacher F. (1998). Defective nitric oxide effector functions lead to extreme susceptibility of *Trypanosoma cruzi*-infected mice deficient in gamma interferon receptor or inducible nitric oxide synthase. *Infect. Immun.* 66 1208–1215. 9488415 10.1128/iai.66.3.1208-1215.1998 PMC108035

[B24] Keating S. M. Deng X. Fernandes F. Cunha-Neto E. Ribeiro A. L. Adesina B. (2015). Inflammatory and cardiac biomarkers are differentially expressed in clinical stages of Chagas disease. *Int. J. Cardiol.* 199 451–459. 10.1016/j.ijcard.2015.07.040 26277551 PMC4868386

[B25] Lewis M. D. Fortes Francisco A. Taylor M. C. Burrell-Saward H. McLatchie A. P. Miles M. A. (2014). Bioluminescence imaging of chronic *Trypanosoma cruzi* infections reveals tissue-specific parasite dynamics and heart disease in the absence of locally persistent infection. *Cell. Microbiol.* 16 1285–1300. 10.1111/cmi.12297 24712539 PMC4190689

[B26] Luz P. R. Miyazaki M. I. Chiminacio Neto N. Padeski M. C. Barros A. C. Boldt A. B. (2016). Genetically determined MBL deficiency is associated with protection against chronic cardiomyopathy in Chagas disease. *PLoS Negl. Trop. Dis.* 10:e0004257. 10.1371/journal.pntd.0004257 26745156 PMC4706301

[B27] Martins-Melo F. R. Ramos A. N. Jr. Alencar C. H. Heukelbach J. (2016). Mortality from neglected tropical diseases in Brazil, 2000-2011. *Bull. World Health Organ.* 94 103–110. 10.2471/BLT.15.152363 26908960 PMC4750431

[B28] Melo R. C. Brener Z. (1978). Tissue tropism of different *Trypanosoma cruzi* strains. *J. Parasitol.* 64 475–482. 10.2307/3279787 96243

[B29] Nagajyothi F. Machado F. S. Burleigh B. A. Jelicks L. A. Scherer P. E. Mukherjee S. (2012). Mechanisms of *Trypanosoma cruzi* persistence in Chagas disease. *Cell. Microbiol.* 14 634–643. 10.1111/j.1462-5822.2012.01764.x 22309180 PMC3556388

[B30] Nogueira L. G. Santos R. H. Ianni B. M. Fiorelli A. I. Mairena E. C. Benvenuti L. A. (2012). Myocardial chemokine expression and intensity of myocarditis in Chagas cardiomyopathy are controlled by polymorphisms in CXCL9 and CXCL10. *PLoS Negl. Trop. Dis.* 6:e1867. 10.1371/journal.pntd.0001867 23150742 PMC3493616

[B31] Paiva C. N. Figueiredo R. T. Kroll-Palhares K. Silva A. A. Silverio J. C. Gibaldi D. (2009). CCL2/MCP-1 controls parasite burden, cell infiltration, and mononuclear activation during acute *Trypanosoma cruzi* infection. *J. Leukoc. Biol.* 86 1239–1246. 10.1189/jlb.0309187 19641038

[B32] Perez A. R. Silva-Barbosa S. D. Berbert L. R. Revelli S. Beloscar J. Savino W. (2011). Immunoneuroendocrine alterations in patients with progressive forms of chronic Chagas disease. *J. Neuroimmunol.* 235 84–90. 10.1016/j.jneuroim.2011.03.010 21496931

[B33] Poveda C. Fresno M. Girones N. Martins-Filho O. A. Ramirez J. D. Santi-Rocca J. (2014). Cytokine profiling in Chagas disease: towards understanding the association with infecting *Trypanosoma cruzi* discrete typing units (a BENEFIT TRIAL sub-study). *PLoS One* 9:e91154. 10.1371/journal.pone.0091154 24608170 PMC3946691

[B34] Rassi A. Jr. Rassi A. Marin-Neto J. A. (2010). Chagas disease. *Lancet* 375 1388–1402. 10.1016/S0140-6736(10)60061-X 20399979

[B35] Requena-Mendez A. Lopez M. C. Angheben A. Izquierdo L. Ribeiro I. Pinazo M. J. (2013). Evaluating Chagas disease progression and cure through blood-derived biomarkers: a systematic review. *Expert Rev. Anti Infect. Ther.* 11 957–976. 10.1586/14787210.2013.824718 24053276

[B36] Rodrigues A. A. Notario A. F. Teixeira T. L. e Silva R. T. Quintal A. P. Alves R. N. (2016). A high throughput analysis of cytokines and chemokines expression during the course of *Trypanosoma cruzi* experimental oral infection. *Acta Trop.* 157 42–53. 10.1016/j.actatropica.2016.01.025 26827742

[B37] Rodrigues A. A. Saosa J. S. da Silva G. K. Martins F. A. da Silva A. A. Souza Neto C. P. (2012). IFN-gamma plays a unique role in protection against low virulent *Trypanosoma cruzi* strain. *PLoS Negl. Trop. Dis.* 6:e1598. 10.1371/journal.pntd.0001598 22509418 PMC3317909

[B38] Roggero E. Perez A. Tamae-Kakazu M. Piazzon I. Nepomnaschy I. Wietzerbin J. (2002). Differential susceptibility to acute *Trypanosoma cruzi* infection in BALB/c and C57BL/6 mice is not associated with a distinct parasite load but cytokine abnormalities. *Clin. Exp. Immunol.* 128 421–428. 10.1046/j.1365-2249.2002.01874.x 12067296 PMC1906265

[B39] Sanoja C. Carbajosa S. Fresno M. Girones N. (2013). Analysis of the dynamics of infiltrating CD4(+) T cell subsets in the heart during experimental *Trypanosoma cruzi* infection. *PLoS One* 8:e65820. 10.1371/journal.pone.0065820 23776551 PMC3679147

[B40] Silva J. S. Twardzik D. R. Reed S. G. (1991). Regulation of *Trypanosoma cruzi* infections in vitro and in vivo by transforming growth factor beta (TGF-beta). *J. Exp. Med.* 174 539–545. 10.1084/jem.174.3.539 1908509 PMC2118925

[B41] Silva J. S. Vespa G. N. Cardoso M. A. Aliberti J. C. Cunha F. Q. (1995). Tumor necrosis factor alpha mediates resistance to *Trypanosoma cruzi* infection in mice by inducing nitric oxide production in infected gamma interferon-activated macrophages. *Infect. Immun.* 63 4862–4867. 7591147 10.1128/iai.63.12.4862-4867.1995 PMC173696

[B42] Soares M. B. Silva-Mota K. N. Lima R. S. Bellintani M. C. Pontes-de-Carvalho L. Ribeiro-dos-Santos R. (2001). Modulation of chagasic cardiomyopathy by interleukin-4: dissociation between inflammation and tissue parasitism. *Am. J. Pathol.* 159 703–709. 10.1016/S0002-9440(10)61741-5 11485928 PMC1850539

[B43] Sousa G. R. Gomes J. A. Fares R. C. Damasio M. P. Chaves A. T. Ferreira K. S. (2014). Plasma cytokine expression is associated with cardiac morbidity in Chagas disease. *PLoS One* 9:e87082. 10.1371/journal.pone.0087082 24603474 PMC3945957

[B44] Talvani A. Rocha M. O. Barcelos L. S. Gomes Y. M. Ribeiro A. L. Teixeira M. M. (2004). Elevated concentrations of CCL2 and tumor necrosis factor-alpha in chagasic cardiomyopathy. *Clin. Infect. Dis.* 38 943–950. 10.1086/381892 15034825

[B45] Taniwaki N. N. Andreoli W. K. Calabrese K. S. da Silva S. Mortara R. A. (2005). Disruption of myofibrillar proteins in cardiac muscle of *Calomys callosus* chronically infected with *Trypanosoma cruzi* and treated with immunosuppressive agent. *Parasitol. Res.* 97 323–331. 10.1007/s00436-005-1429-0 16075261

[B46] Torrico F. Heremans H. Rivera M. T. Van Marck E. Billiau A. Carlier Y. (1991). Endogenous IFN-gamma is required for resistance to acute *Trypanosoma cruzi* infection in mice. *J. Immunol.* 146 3626–3632. 1902858

[B47] Udoko A. N. Johnson C. A. Dykan A. Rachakonda G. Villalta F. Mandape S. N. (2016). Early regulation of profibrotic genes in primary human cardiac myocytes by *Trypanosoma cruzi*. *PLoS Negl. Trop. Dis.* 10:e0003747. 10.1371/journal.pntd.0003747 26771187 PMC4714843

[B48] WHO (2017). *Chagas Disease (American Trypanosomiasis)*. http://www.who.int/mediacentre/factsheets/fs340/en/

[B49] Yoshida N. (1983). Surface antigens of metacyclic trypomastigotes of *Trypanosoma cruzi*. *Infect. Immun.* 40 836–839. 6341250 10.1128/iai.40.2.836-839.1983 PMC264932

[B50] Zingales B. Andrade S. G. Briones M. R. Campbell D. A. Chiari E. Fernandes O. (2009). A new consensus for *Trypanosoma cruzi* intraspecific nomenclature: second revision meeting recommends TcI to TcVI. *Mem. Inst. Oswaldo Cruz* 104 1051–1054. 10.1590/S0074-02762009000700021 20027478

[B51] Zweifel M. Matozan K. Dahinden C. Schaffner T. Mohacsi P. (2010). Eotaxin/CCL11 levels correlate with myocardial fibrosis and mast cell density in native and transplanted rat hearts. *Tansplant. Proc.* 42 2763–2766. 10.1016/j.transproceed.2010.05.152 20832583

